# Comparison of acupotomy release combined with glucocorticoid intrathecal injection versus isolated glucocorticoid intrathecal injection for carpal tunnel syndrome: A protocol for a meta-analysis of comparative studies

**DOI:** 10.1097/MD.0000000000032940

**Published:** 2023-03-03

**Authors:** Yukun Liu, Jingfan Yang, Wei Dong, Weitong Liu, Li Chen, Jiao Li, Jiankun Chen, Xing Zhou, Hong Yin, Jinlei Li

**Affiliations:** a Anning Municipal Hospital of Traditional Chinese Medicine, Anning City, China; b Kunming Municipal Hospital of Traditional Chinese Medicine, Kunming City, Yunnan Province, China; c The First Affiliated Hospital of Zhejiang Chinese Medical University, Hangzhou City, Zhejiang, China; d Kunming University of Science and Technology Hospital, Kunming City, Yunnan Province, China.

**Keywords:** acupotomy release, carpal tunnel syndrome, glucocorticoid intrathecal injection, protocol, small needle knife

## Abstract

Carpal tunnel syndrome (CTS) is more common in people who need to engage in repetitive wrist work. Once it has occurred, localized pain and numbness of the fingers will develop, in severe cases, muscle atrophy will even arise, and after rest and physical therapy, many patients will still be unable to relieve or recur. In this case, the patient can receive intrathecal glucocorticoid injections, but the hormone injections alone can only provide short-term relief, and because the mechanical factors of median nerve compression are not fundamentally removed. Therefore, combined acupotomy release can help to release the compression of the transverse carpal ligament on the nerve and increase the volume within the carpal tunnel to achieve more satisfactory long-term results. Hence, a meta-analysis is necessary to provide evidence whether there is a significant difference in the treatment of CTS with acupotomy release combined with glucocorticoid intrathecal injection (ARGI) compared with isolated glucocorticoid intrathecal injection (GI).

**Methods:**

We will search, with no time restriction, without any restriction of language and status, the time from the establishment of the database to October 2022, on the following databases: PubMed, Cochrane central register of controlled trials, Web of Science, Chinese national knowledge infrastructure, Wanfang data, Chinese scientific journals database, Chinese databases SinoMed, and electronic databases. The electronic database search will be supplemented by a manual search of the reference lists of included articles. We will apply the risk-of-bias tool of the Cochrane collaboration for randomized controlled trials to assess the methodological quality. Risk-of-Bias Assessment Tool for nonrandomized studies was used to evaluate the quality of comparative studies. Statistical analysis will be conducted using RevMan 5.4 software.

**Results:**

This systematic review will evaluate the difference in efficacy of ARGI versus isolated GI in the treatment of CTS.

**Conclusion:**

The conclusion of this study will provide evidence for judging whether ARGI is superior to GI for treatment of CTS.

## 1. Introduction

Carpal tunnel syndrome (CTS) is the most widespread peripheral nerve entrapment disorder,^[1]^ which mainly results from decreased volume and increased pressure in the carpal tunnel, producing median nerve entrapment and consequently clinical symptoms mainly pain, abnormal sensation, and limited hand function.^[2,3]^ These will seriously affect the quality of daily life of patients.^[4]^ CTS, as one of the common orthopedic diseases, epidemiological studies have found that the adult prevalence is 3% to 6%, the patient group is more common in women, 2 to 4 times more than men, and the peak age of onset is between 45 and 54 years.^[5–7]^ With reference to the severity of the patient symptoms and the duration of the disease, different treatment plans can be developed, and most patients can get significant relief with conservative treatment, of which glucocorticoid intrathecal injection is the classical treatment plan,^[8]^ but the 2014 European CTS treatment guidelines recommend that closed treatment should not exceed 3 times and the interval between 2 closed treatments is 2 to 3 months.^[9]^ Meanwhile, the Chinese traditional treatment method represented by small needle knife has also achieved satisfactory results in the treatment of carpal tunnel syndrome, which can effectively release the transverse carpal ligament to achieve the effect of releasing carpal tunnel pressure and median nerve compression.^[10,11]^

The vast majority of patients with nonrecalcitrant carpal tunnel syndrome can be treated satisfactorily with conservative rather than surgical treatment.^[12–14]^ After adequate rest, functional rehabilitation and oral medication, intrathecal glucocorticoid injection is recommended as the first choice,^[15]^ which can effectively reduce the local inflammatory response and median nerve edema, thus achieving symptomatic relief.^[16,17]^ However, some scholars believe that this can only delay the symptoms in the short-term and cannot solve the problem at the root, because mechanical compression of the median nerve does not achieve relaxation by hormone injection alone.^[18]^ At this point, combining acupotomy release the transverse carpal ligament, release the pressure of the carpal tunnel, and free the compressed median nerve is more effective in enhancing clinical satisfaction and reducing the probability of recurrence.^[19–22]^

With the recent in-depth study of this research by a wide range of scholars, more and more relevant literature is presented, and there is no high-quality evidence-based medical evidence to guide clinical decision makers, so we attempted to conduct a meta-analysis of the literature in this field to evaluate and compare the functional effects and complication rates of acupotomy release combined with glucocorticoid intrathecal injection versus isolated glucocorticoid intrathecal injection for CTS, but hopefully the conclusions we reached can provide a reliable reference for future treatment options.

## 2. Methods

### 2.1. Study registration

We have prospectively registered this research at the international prospective register of systematic reviews (PROSPERO)-Registration number: CRD42022373913. We performed this protocol based on the preferred reporting items for systematic review and meta-analysis protocols (PRISMA-P) statement guidelines.^[23]^

### 2.2. Inclusion criteria

#### 2.2.1. Type of participants.

Only studies in which patients have a confirmed clinical diagnosis of carpal tunnel syndrome will be included regardless their country, ethnicity, sex, and occupation and educational status. Fracture and dislocation, muscle injury, bone tuberculosis, bone tumors, or any systematic diseases will be excluded.

#### 2.2.2. Type of interventions.

Intervention of the experimental group must cover acupotomy release combined with glucocorticoid intrathecal injection. There will be no limitations on the needle materials, treatment methods and treatment courses. The control group must cover glucocorticoid intrathecal injection.

#### 2.2.3. Type of outcome measurements.

##### 2.2.3.1. Primary outcomes.

The primary outcome are effectiveness and Boston carpal tunnel questionnaire.

##### 2.2.3.2. Secondary outcomes.

The secondary outcome are visual analogue score, recurrence rates, quality of life and adverse events, such as hemorrhage, serious discomfort, abscess, subcutaneous nodules, and infection.

#### 2.2..4. Type of studies.

We will include comparative studies which published in Chinese or English, such as randomized controlled trials, retrospective studies and cohort studies. Review, case reports, experimental studies, expert experience, and animal studies and conference abstracts will be excluded.

### 2.3. Search strategy

CNKI, Wanfang, VIP, CBM, PubMed, Embase, and Cochrane Library databases were searched for this study. The search string is built as follows: (carpal tunnel syndrome OR Median nerve) AND (acupotomy OR small acupotomy OR needle knife). The search strategy in PubMed is shown in Table 1. In addition, the reference lists of previously published systematic reviews of carpal tunnel syndrome were manually examined for further pertinent studies.

**Table 1:** PubMed database search strategy.

Search number	Items
1	“ carpal tunnel syndrome “[Mesh]
2	carpal tunnel syndrome [Title/Abstract]
3	median nerve [Title/Abstract]
4	1 OR 2 OR 3
5	acupotomy [Title/Abstract]
6	small acupotomy [Title/Abstract]
7	needle knife [Title/Abstract]
8	5 OR 6 OR 7
9	4 AND 8

### 2.4. Study selection

**Figure 1.**
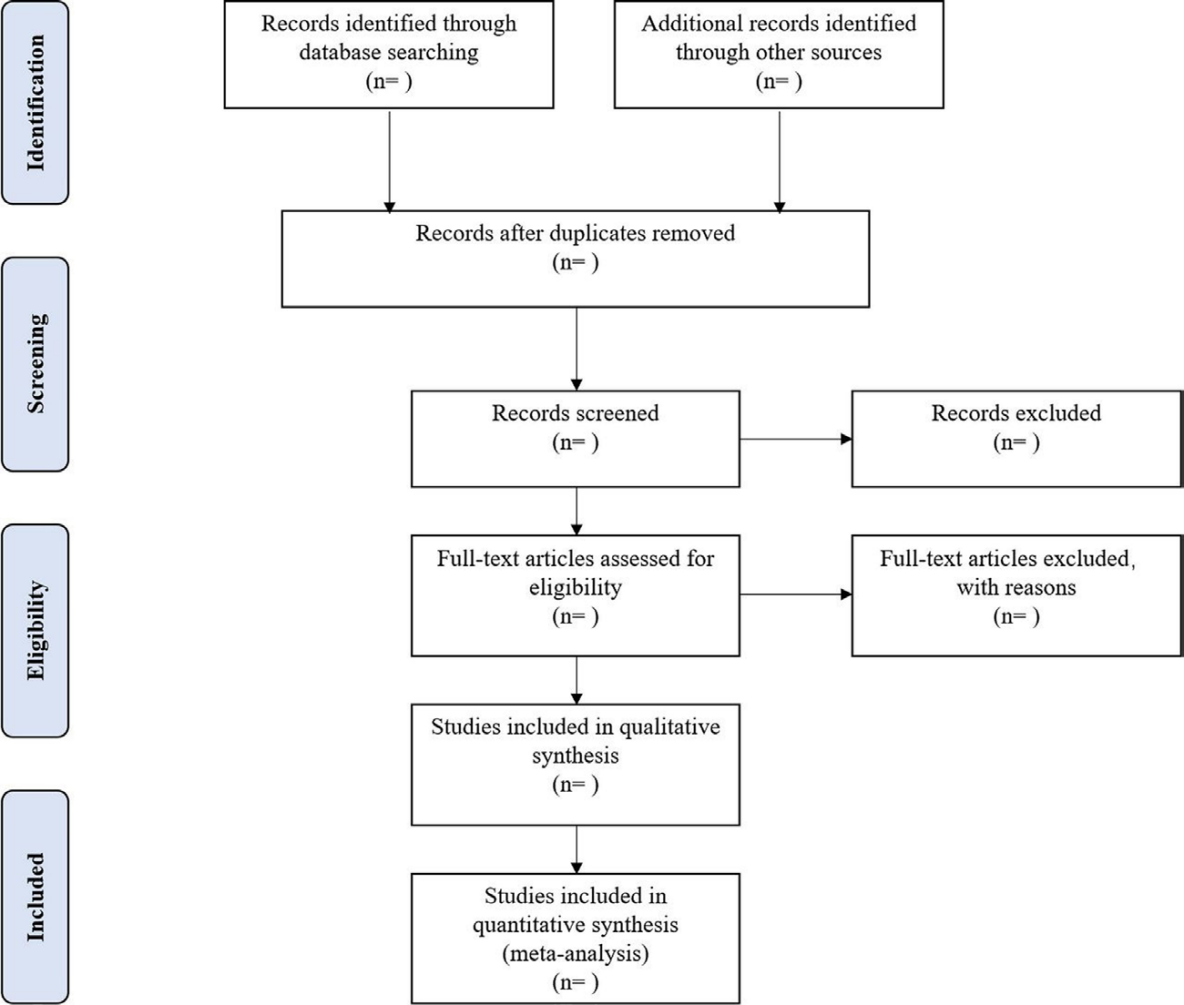
Flowchart of literature selection.

Different researchers will separately screen the titles and abstracts of records acquired potential eligibility which comes from the electronic databases. The obtained literature is managed by Noto express, irrelevant and duplicate articles are excluded by reading the title and abstract, Full texts screening and data extraction will be conducted afterward independently, and finally selected according to the inclusion criteria, Any disagreement will be resolved by discussion with the third author until consensus is reached or by consulting a third author. PRISMA-P flowchart Will be used to show the selection procedure (Fig. 1).

### 2.5. Data extraction and management

The following data were extracted: lead author; publication year; country of origin; study design; sample size; age; interventions of experimental groups and control groups; outcome measures and complications. Any differences of opinion will be resolved through group discussion or consultation with a third reviewer. When relevant data is not reported, we will contact the author via email or other means to obtain missing data. The PRISMA-P flow diagram will be filled out after the screening study is completed to provide specific information.

### 2.6. Risk-of bias assessment

Two independent investigators evaluated the quality of the included studies. The Cochrane collaboration risk-of bias tool was used to evaluate the quality of the randomized controlled trials. The methodological quality of the nonrandomized studies was assessed using the risk-of-bias Assessment Tool for nonrandomized studies. The level of evidence was assessed according to the Oxford Centre for evidence-based medicine levels of evidence.

### 2.7. Data synthesis

Statistical analysis will be conducted using RevMan 5.4 software (Cochrane collaboration). The mean difference will be used as the effect analysis statistic for continuous variables, while the risk ratio will be used as the effect analysis statistic for categorical variables. We will also calculate 95% confidence interval for each statistic, summarize statistical heterogeneity among summary data using the *I*^2^ statistic. Cases with *I*^2^ ≤ 50% will not be considered to have significant heterogeneity, thus a fixed-effects model will be applied for meta-analysis. In cases where there is statistical heterogeneity among studies, we will further analyze the source of heterogeneity. A random-effects model will be used to pool the data, after excluding the obvious source of clinical heterogeneity, and in cases where obvious clinical heterogeneity exists, the researchers will perform subgroup, sensitivity or only descriptive analyses. Study-specific and pooled estimates will be graphically presented using forest plots, *P* < .05 considered statistically significant.

### 2.8. Subgroup analysis

If there is significant heterogeneity in the included trials, we will perform a subgroup analysis based on the severity of CTS and the type of control intervention.

### 2.9. Sensitivity analysis

Sources of heterogeneity were assessed by sensitivity analysis, by excluding studies of low quality or small sample size, if the heterogeneity did not change significantly, and the results were robust. otherwise, the excluded studies may have been source of heterogeneity.

### 2.10. Publication bias

In this study, fewer than 10 included studies were evaluated for publication bias using funnel plot, otherwise Egger regression test would be used.^[24,25]^

### 2.11. Ethics and dissemination

No ethical approval is required because the study will be a review of literature and will not obtain data from a single patient. We will publish our findings through a peer-reviewed journal.

## 3. Discussion

The objective of this study was to comparatively assess the efficacy and complications associated with acupotomy release combined with glucocorticoid intrathecal injection and isolated glucocorticoid intrathecal injection in the treatment of CTS. The use of conservative treatment strategies is more effective in relieving symptoms and reducing the rate of trauma and surgery in patients with CTS. This study will integrate the most recent and comprehensive clinical evidence in the field, with the hope of providing useful, high-grade evidence-based medicine for patients and clinicians.

## Author contributions

**Conceptualization:** Yukun Liu, Wei Dong, Jinlei Li.

**Formal analysis:** Weitong Liu.

**Funding acquisition:** Jingfan Yang, Jinlei Li.

**Investigation:** Li Chen.

**Methodology:** Yukun Liu.

**Project administration:** Wei Dong.

**Resources:** Jiao Li.

**Software:** Xing Zhou.

**Supervision:** Jingfan Yang, Jiankun Chen.

**Validation:** Hong Yin.

**Visualization:** Jingfan Yang.

**Writing – original draft:** Yukun Liu, Jinlei Li.
